# Mode of Action of *1*-Naphthylphthalamic Acid in Conspicuous Local Stem Swelling of Succulent Plant, *Bryophyllum calycinum*: Relevance to the Aspects of Its Histological Observation and Comprehensive Analyses of Plant Hormones

**DOI:** 10.3390/ijms22063118

**Published:** 2021-03-18

**Authors:** Agnieszka Marasek-Ciolakowska, Michał Dziurka, Urszula Kowalska, Justyna Góraj-Koniarska, Marian Saniewski, Junichi Ueda, Kensuke Miyamoto

**Affiliations:** 1 The National Institute of Horticultural Research, Konstytucji 3 Maja 1/3, 96-100 Skierniewice, Poland; urszula.kowalska@inhort.pl (U.K.); justyna.goraj@inhort.pl (J.G.-K.); marian.saniewski@inhort.pl (M.S.); 2 The Franciszek Górski Institute of Plant Physiology, Polish Academy of Sciences, Niezapominajek 21, 30-239 Kraków, Poland; m.dziurka@ifr-pan.krakow.pl; 3 Department of Biological Science, Graduate School of Science, Osaka Prefecture University, 1-1 Gakuen-cho, Naka-ku, Sakai, Osaka 599-8531, Japan; ueda@b.s.osakafu-u.ac.jp; 4 Faculty of Liberal Arts and Sciences, Osaka Prefecture University, 1-1 Gakuen-cho, Naka-ku, Sakai, Osaka 599-8531, Japan

**Keywords:** crassulaceae, cytokinin, hormonal crosstalk, indoleacetic acid, jasmonic acid, polar auxin transport, stem swelling, succulent plant

## Abstract

The mode of action of *1*-naphthylphthalamic acid (NPA) to induce conspicuous local stem swelling in the area of its application to the growing internode in intact *Bryophyllum calycinum* was studied based on the aspects of histological observation and comprehensive analyses of plant hormones. Histological analyses revealed that NPA induced an increase in cell size and numerous cell divisions in the cortex and pith, respectively, compared to untreated stem. In the area of NPA application, vascular tissues had significantly wider cambial zones consisting of 5–6 cell layers, whereas phloem and xylem seemed not to be affected. This indicates that stem swelling in the area of NPA application is caused by stimulation of cell division and cell enlargement mainly in the cambial zone, cortex, and pith. Comprehensive analyses of plant hormones revealed that NPA substantially increased endogenous levels of indole-3-acetic acid (IAA) in the swelling area. NPA also increased endogenous levels of cytokinins, jasmonic acid, and its precursor, 12-oxo-phytodienoic acid, but did not increase abscisic acid and gibberellin levels. It was shown, using radiolabeled ^14^C-IAA, that NPA applied to the middle of internode segments had little effect on polar auxin transport, while 2,3,5-triiodobenzoic acid substantially inhibited it. These results strongly suggest that NPA induces changes in endogenous levels of plant hormones, such as IAA, cytokinins, and jasmonic acid, and their hormonal crosstalk results in a conspicuous local stem swelling. The possible different mode of action of NPA from other polar auxin transport inhibitors in succulent plants is extensively discussed.

## 1. Introduction

Plant growth, such as cell elongation, cell division, and cell enlargement, is primarily regulated by plant hormones and other plant growth substances. Among plant hormones, naturally occurring auxin, indole-3-acetic acid (IAA), mainly synthesized in the apical part of shoots and young leaves, shows a specific basipetal movement known as polar auxin transport. In this process, auxin is transported basipetally between cells toward the root tip through the stele in the aboveground parts, and then redirected symmetrically in specific root tip cells, and transported back towards the root elongation zone [1,2,3,4,5,6,7]. Commonly used polar auxin transport inhibitors such as *1*-naphthylphthalamic acid (NPA), 2,3,5-triiodobenzoic acid (TIBA) and morphactins (methyl 2-chloro-9-hydroxyfluorene-9-carboxylic acid, IT 3456, and 9-hydroxyfluorene-9-carboxylic acid, HFCA) have been demonstrated to show various effects by interfering with polar auxin transport and changing auxin distribution, and in consequence, to perturb auxin-related plant growth and development like root formation, cell elongation, and tropisms (see review by Ueda et al. [6]).

Recently, NPA was found to exhibit unexpected physiological effects in succulent plants: NPA did not inhibit root formation in shoot cuttings of *Bryophyllum calycinum*, *B. daigremontianum*, *Kalanchoe tubiflora*, and *K. blossfeldiana*, while TIBA and morphactins (IT 3456 and HFCA) completely inhibited it in these species [7]. NPA also did not inhibit root development on plantlets formed on leaves excised from intact *B. marnierianum*, acting in the opposite way to TIBA [8]. In addition, unlike TIBA and HFCA, NPA did not affect the induction of epinasty and petiole bending induced by exogenously applied IAA in intact and detached *B*. *calycinum* leaves [9,10]. We also found that NPA induced conspicuous local stem swelling only in the area of its application in intact plants of *B. calycinum*, and in the decapitated ones when IAA was applied simultaneously on the top of the internode [11].

In contrast, the application of TIBA [11] and HFCA (Saniewski et al. [11], unpublished results) in the same manner to *B. calycinum* plants did not induce conspicuous local stem swelling, whereas slight swelling along the entire internode above the treatment area was observed. These results suggest that the mode of actions of NPA is different from that of TIBA and HFCA in species of the family Crassulaceae, and that stem swelling in *B. calycinum* is caused by the interaction with NPA and IAA.

However, the mechanism by which NPA induces conspicuous local stem swelling has remained unclear. The purpose of this study was to clarify the mechanism of NPA stimulating effect on local stem swelling in *B. calycinum* based on the aspects of its histological observations and comprehensive analyses of plant hormones. The relationships between NPA and polar auxin transport in stem swelling and rooting of shoot cuttings in succulent plants are also discussed.

## 2. Results

### 2.1. Effect of NPA on Conspicuous Local Stem Swelling in Succulent Plants

**Figure 1:**
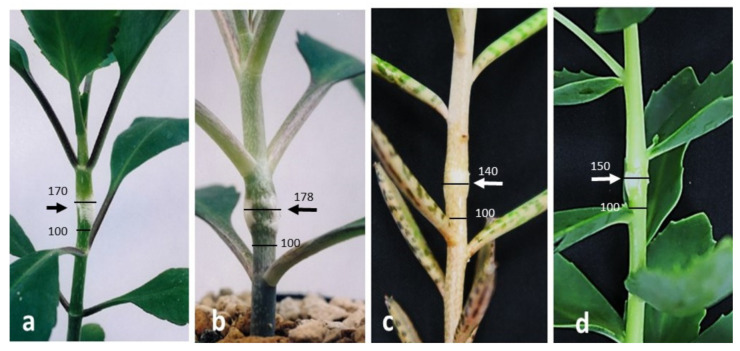
Conspicuous local stem swelling in succulent plants induced by NPA (0.4%, *w/w* in lanolin) after 8 days of treatment. (**a**) *Bryophyllum calycinum,* (**b**) *B. daigremontianum*, (**c**) *Kalanchoe tubiflora*, and (**d**) *Sedum spectabile.* Arrows indicate the site of NPA application as a 2-mm-wide ring of lanolin paste around the stem in the intensively growing internode. Values with lines in the photographs indicate relative stem diameter (%) to the non-treated area (100%) based on the photographs.

As described in the Introduction, we have reported that NPA induced conspicuous local stem swelling only at the site of its application in intact plants and in the decapitated internode of *B. calycinum* when IAA was applied simultaneously on the top of the internode [11]. It should be mentioned that such conspicuous local stem swelling was not induced by the application of TIBA [11] or HFCA (Saniewski et al. [11], unpublished results). As shown in Figure 1, conspicuous local stem swelling induced by NPA was confirmed in the present study. NPA substantially induced conspicuous local stem swelling also in some other succulent plants, such as *B. daigremontianum*, *K. tubiflora*, and *Sedum spectabile* (Figure 1)*,* but TIBA did not show that effect (see Saniewski et al. [11]). This fact suggests that, differently from TIBA, NPA induces conspicuous local stem swelling as an unusual mode of action regarding some auxin-regulated process in succulent plants.

In our previous study, rooting of *B. calycinum*, *B. daigremontianum*, *K. tubiflora*, and *K. blosfeldiana* shoot cuttings was inhibited by the application of TIBA and morphactin (IT 3456), however, not by NPA [7]. As shown in Figure S1 and Table S1, shoot cuttings of *S. spectabile* easily rooted and developed a root system well, when they were incubated both in water and soil medium. The application of TIBA (0.2%, *w/w*) or HFCA (0.2%, *w/w*) almost completely inhibited root formation and shoot growth. In contrast, NPA (0.4%, *w/w*) application neither inhibited root formation nor shoot growth in the cuttings, regardless of growth conditions. These results strongly support the hypothesis that NPA shows unusual effects unlike other polar auxin transport inhibitors in succulent plants.

### 2.2. Histological Analyses of Conspicuous Local Stem Swelling in Succulent Plants

**Figure 2:**
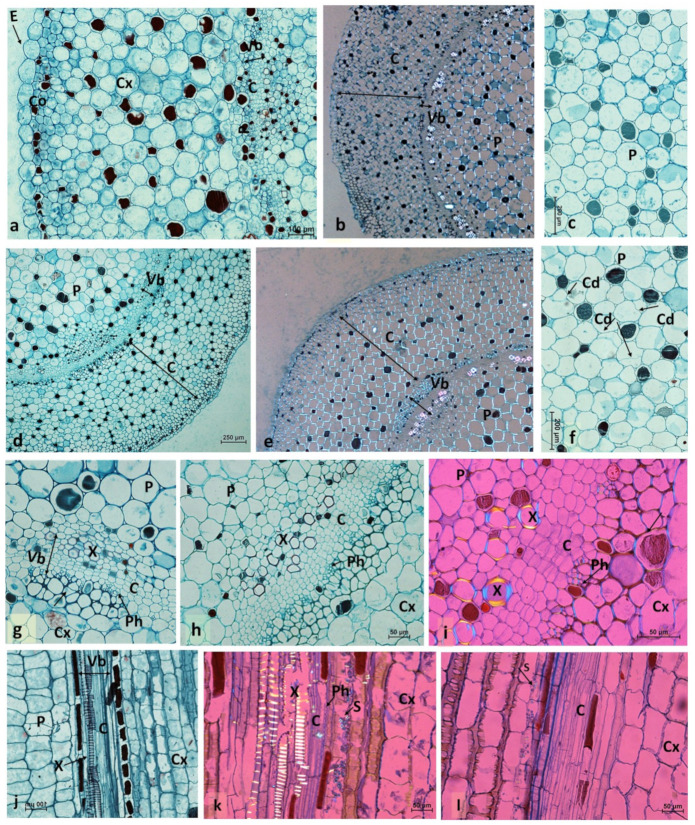
Anatomical details in the middle of *B. calycinum* internode. (**a**–**c**): Cross-section of the Scheme 0. *w/w*). (**d**): Cross-section under LM. (**e**): Cross-section under LMP. (**f**): Fragment of the pith in LM. (**g**–**i**): Details of vascular tissue on the cross-section of the control stem under LM (**g**) and LMP (**h**), and of stem swelling induced by NPA application under LMP (**i**). (**j**–**l**): Details of vascular tissue on the longitudinal section of the control stem under LM (**j**) and LMP (**k**), and of conspicuous local stem swelling induced by NPA under LMP (**l**). C: cambium; Cd: cell division; Co: collenchyma; Cx: cortex; E: epidermis; P: pith; Ph: phloem; S: starch; VB; vascular tissues; X: xylem.

Figure 2 shows anatomical observations of *B. calycinum* stem eight days after treatment with or without NPA (0.4%, *w/w* in lanolin).

The stem surface of the control plants was covered by large epidermal cells (E) and several layers of collenchyma cells (Cx) (Figure 2a). Both cortex and pith consisted of large, vacuolated collenchymatous cells, whose cell walls glowed in the polarized light microscopy image (LMP) (Figure 2a–c). In *B. calycinum*, vascular tissue was found at the border between cortex and pith. Cambium formed a ring consisting of 2–3 cell layers surrounded by a cluster of phloem elements from the side of cortex and a cluster of xylem elements from the side of pith (Figure 2b,g).

The effect of NPA on conspicuous local stem swelling is shown in Figure 2d–f,h,i,l. An extreme increase in cortex cell size was observed (Figure 2e) compared to control; moreover, numerous cell divisions were observed in pith (Figure 2f). At the site of NPA treatment, vascular tissue had a significantly wider cambial zone consisting of 5–6 cell layers, whereas phloem and xylem seemed not to be affected by NPA treatment (Figure 2h,i,l). All these processes led to an increase in stem size, resulting in conspicuous local stem swelling.

Microscopic observations were also carried out on *K. tubiflora* (Figure S2) and *S. spectabile* (Figure S3) to analyze the processes of local stem swelling formation induced by NPA (0.4%, *w/w* in lanolin).

As shown in Figure S2 in *K. tubiflora,* in comparison to control, numerous cell divisions were observed in the cortex of NPA-treated stem, and their cell walls were formed in different directions or in both periclinal and anticlinal directions, resulting in an increase in the number of cortex cells. Moreover, significant differences between control and NPA-treated plants were observed in vascular tissues. In control plants, cambium consisted of 3–4 cell layers, and xylem elements glowed in polarized light, while vascular tissue in NPA-treated plants had a significantly wider cambial zone consisting of approximately 6–7 cell layers, and no glowing ring of xylem cells was observed in the conspicuous local swelling area under polarized light. Anticlinal cell divisions were observed in cambium of NPA-treated plants.

Numerous cell divisions and an increase in the size of cortex cells were observed in *S. spectabile* stem treated with NPA compared to control (Figure S3). A significant increase in the size of parynchymatous cells was also observed in the pith. At the site of NPA treatment, vascular tissues had significantly wider cambial and xylem zones compared to control. A higher number of cambium layers was also observed in the longitudinal section. All these processes in *S. spectabile* led to conspicuous local stem swelling.

All the above processes led to an increase in stem size in *K. tubiflora*, *S. spectabile*, and *B. calycinum*, resulting in conspicuous local stem swelling.

### 2.3. Endogenous Levels of Plant Hormones in Relation to NPA-Induced Conspicuous Local Stem Swelling in Succulent Plants

**Figure 3:**
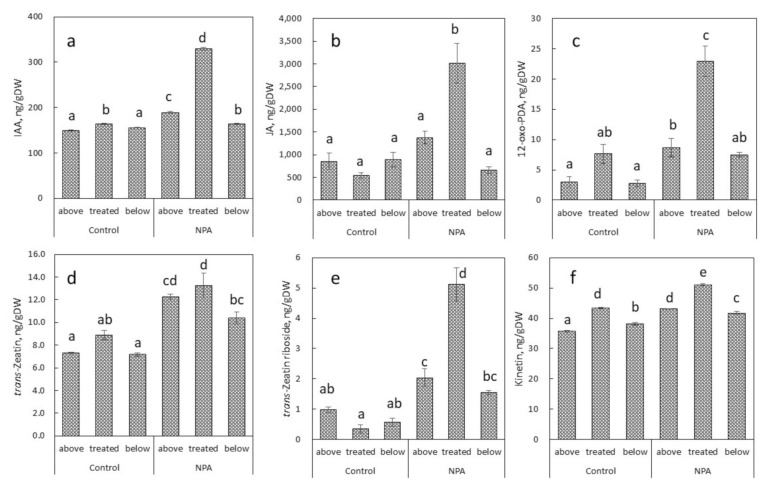
Effect of NPA (0.4%, *w/w* in lanolin) on endogenous levels of IAA (**a**), jasmonic acid (JA) (**b**), 12-oxo-phytodienoic acid (12-oxo-PDA) (**c**), *trans*-zeatin (**d**), *trans*-zeatin riboside (**e**), and kinetin (**f**) in 4–5-mm pieces of internode at the NPA treatment site, and areas above and below in *B. calycinum.* Treated, above and below in the figure indicate plant hormone levels at, above and below areas of the treatment, respectively. Values are means with standard errors (*n* = 3). Different letters indicate statistical differences in Duncan’s multiple range test at *p* < 0.05 after analysis of variance (ANOVA).

Eight days after treatment with or without NPA (0.4%, *w/w* in lanolin), endogenous levels of plant hormones in *B. calycinum* and *B. daigremontianum* were investigated in 4–5-mm internode segments at the treatment site, and in the areas above and below the treatment (Figure 3 and Figure S4, and Table 1 and Table S2).

IAA was successfully identified in the control stem treated only with lanolin for 8 days. The application of lanolin alone had no effect on IAA level at the site, and the areas above and below the treatment in *B. calycinum*. On the other hand, NPA treatment significantly increased endogenous IAA levels only in the area of stem swelling, but not in the areas above and below the treatment (Figure 3a).

Endogenous jasmonic acid (JA) and 12-oxo-phytodienoic acid (12-oxo-PDA), an important precursor of JA, were also detected in *B. calycinum* stem, whereas 12-oxo-PDA was present at low concentrations (Figure 3b,c). Increased endogenous levels of these compounds were especially detected at the area of NPA treatment in comparison to the areas above and below the treatment. Endogenous level of salicylic acid (SA) was also increased by NPA treatment (Table 1). Until now, there is no available data about the connection of NPA on the metabolism of SA in plants. On the other hand, it is well known that SA is an inhibitor of ethylene and JA biosynthesis.

Five cytokinins, *trans*-zeatin, *cis*-zeatin, *trans*-zeatin riboside, *cis*-zeatin riboside, and kinetin (KN), were found in *B. calycinum* stem. Endogenous levels of *trans*-zeatin, *trans*-zeatin riboside, and kinetin were substantially increased by NPA treatment, especially at the area of stem swelling (Figure 3d–f). Endogenous levels of *cis*-zeatin and *cis*-zeatin riboside were almost the same in all areas tested in the control plants and NPA application slightly increased these levels (Table 1).

On the other hand, only small differences in abscisic acid (ABA) levels were found between the swelling area of the stem induced by NPA and the areas above and below the treatment (Table 1).

Gibberellins (GAs), GA_1_, GA_4_, GA_8_, GA_9_, GA_13_, GA_19_, GA_44_, and GA_53_ were detected in the stem (Table 1). GA_8_, GA_9_, GA_19_, GA_53_, and GA_1_ occurred at the highest levels and the concentrations of GA_15_, GA_4_, and GA_44_ were low. NPA treatment had little effect on the levels of these GAs.

Increased levels of IAA, JA and 12-oxo-PDA, SA, *trans*-zeatin riboside, and *cis*-zeatin riboside were observed in *B*. *daigremontianum* treated with NPA, and they were similar to those in *B. calycinum* (Figure S4 and Table S2).

The results described above indicate that the application of NPA to plant species of the genus *Bryophyllum* induces changes in endogenous levels of several plant hormones.

### 2.4. Effect of NPA on Polar Auxin Transport in Bryophyllum Calycinum Stem Segments

**Figure 4:**
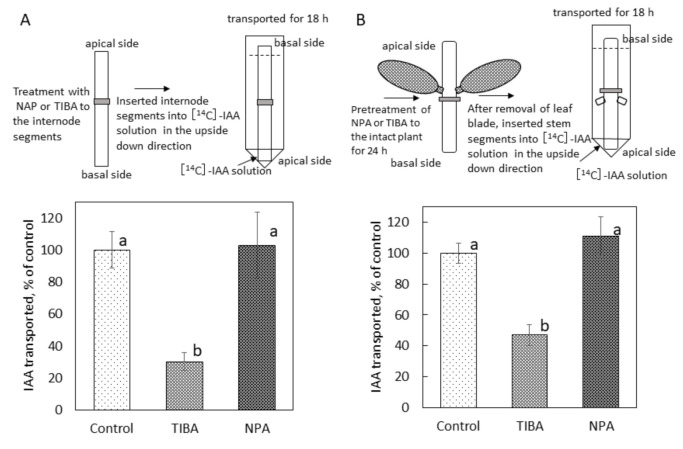
Effect of NPA and TIBA on polar auxin transport in stem segments of *B. calycinum*. (**A**): Internode segments (25 mm in length) were prepared, and then, lanolin with or without NPA (0.4%, *w/w* in lanolin) or TIBA (0.4%, *w/w* in lanolin) was applied to the middle of the internode. (**B**)**:** Internode segments (40 mm in length) were prepared from intact stems pretreated with TIBA or NPA for 24 h prior to the experiment. Internode segments treated only with lanolin served as control segments. After removing leaf blade, the stem segments were placed into vials facing down the apical side. Polar auxin transport was assessed by radioactivity of [^14^C]-IAA transported from the apical to the basal end of the stem segments for 18 h. The results were expressed as percentages of control values. Vertical lines represent SE of the mean (*n* = 3). Different letters indicate statistical difference in Student’s *t*-test at *p* < 0.05 after ANOVA.

The effect of NPA on polar auxin transport in *B. calycinum* internode was investigated using radiolabeled ^14^C-indole-3-acetic acid ([1-^14^C]IAA) and compared to that of TIBA. Figure 4A shows that NPA applied at the middle of the internode segment as a lanolin paste did not inhibit polar auxin transport, whereas TIBA significantly inhibited it. NPA pretreatment of intact plants for 24 h prior to the polar auxin transport experiment showed no effect on polar auxin transport, but TIBA significantly inhibited it (Figure 4B).

## 3. Discussion

As described in the Introduction, inhibitors of polar auxin transport such as NPA, TIBA, and morphactins (IT 3456, HFCA) were expected to perturb plant growth and development (see review by Ueda et al. [6]). NPA induced conspicuous local stem swelling in succulent plants. Unlike NPA, TIBA and morphactin (IT 3456, HFCA) application to the internode of intact *Bryophyllum calycinum* plants did not induce such conspicuous local swelling, while they induced slight swelling along the entire stem above the treated area ([11]; Saniewski et al., unpublished results). It is important to elucidate the mode of novel physiological action of NPA in conspicuous local stem swelling for stiffening of plants, food production, and its industry [12,13].

As shown in Figure 1, our previous results [11] that NPA application as a lanolin paste induced conspicuous local stem swelling of *B. calycinum* were confirmed in the current study. Such NPA-induced conspicuous local stem swellings were commonly observed in other genera of succulent plants, e.g., *Kalanchoe* and *Sedum*. This observation was true for the rooting of shoot cuttings of succulent plants (Figure S1 and Table S1). The application of NPA to *B. calycinum* shoot cuttings did not suppress adventitious root formation of shoot cuttings, as previously reported by Saniewski et al. [7]. This lack of NPA effect on rooting was observed in shoot cuttings of *B. daigremontianum*, *K. tubiflora* and *K. blossfeldiana,* and *S. spectabile*, while TIBA, morphactin 3456, and HFCA completely inhibited it in these species. These results strongly suggest that, unlike TIBA and HFCA, NPA does not inhibit polar auxin transport in succulent plants.

The application of NPA to the middle area of the stem did not affect polar auxin transport in *B. calycinum,* but TIBA significantly inhibited it, even when the stem was pretreated for 24 h with NPA prior to the bioassay-driven polar auxin transport experiment (Figure 4). The reason of this apparent discrepancy in the mode of action of NPA and other inhibitors of polar auxin transport (TIBA and morphactins) on rooting, conspicuous local stem swelling and polar auxin transport in succulent plants has not yet been elucidated; however, different interaction with other proteins relevant to polar auxin transport is possible and cannot be excluded [6,7]. Based on the study involving transcript, protein, and metabolite temporal dynamics in the Crassulacean acid metabolism (CAM) plant *Agave* [14], along with the fact that CAM plants are different from C3 plants in photosynthetic mechanisms, it cannot be excluded that NPA binding and/or associated proteins in *B. calycinum* are not the same as in C3 plants such as *Arabidopsis.* As opposed to TIBA, NPA hardly inhibited hypocotyl rhizogenesis of the CAM plant *Mesembryanthemum crystallinum* [15]. TIBA increased root bud growth in the CAM plant *Euphorbia escula* [16], but NPA had little effect on root bud growth [17]. NPA did not affect the formation of vegetative structures at bracteoles, which are formed after flower bud removal in *Agave tequilana* [18]. Considering these facts, different effects of NPA from other polar auxin transport inhibitors are believed to be due to the ineffectiveness or lesser effect of NPA on polar auxin transport in the stem of succulent plants. Different subsets of auxin transport molecules may be involved in the unusual effect of NPA compared to TIBA.

Histological analyses of conspicuous local stem swelling induced by NPA revealed that a marked increase in cortex cell size, numerous cell divisions of pith cells, and a significantly wider cambial zone consisting of 5–6 cell layers were observed as compared to the untreated stem (Figure 2). Stimulation of cambium activity by NPA was commonly observed in other species, e.g., *K. tubiflora* (Figure S2) and *S. spectabile* (Figure S3), as well as the induction of local stem swelling. Moreover, numerous cell divisions were observed in the cortex and pith, accompanied either by an increase in cell size or a change in the shape. The reaction to NPA treatment of other elements of vascular tissues varied depending on the species, and the phloem and xylem in *B. calycinum* seemed not to be affected. In *S. spectabile*, enlargement of the xylem zone was observed, while the loss of lignification by some xylem elements was manifested in *K. tubiflora* as a lack of cell illuminance under LMP. These results suggested that the common feature, i.e., stimulation of cambium activity and enlargement of this zone, multiple cell divisions of cortex and pith, accompanied by an increase in cell size or change of shape in NPA treatment, led to an increase in stem size, resulting in conspicuous local stem swelling.

Auxin is a factor that triggers cell divisions in plant cultures, including the tobacco (*Nicotiana tabacum* cv. Virginia Bright Italia) VBI-0 cell line [19]. Campanoni et al. [19] showed that NPA at a concentration of 5 µM, at which auxin transport is impaired but not completely eliminated, did not reduce the overall rate of cell divisions and viability; however, the distribution of cell divisions was strongly altered without affecting cell elongation. NPA at a concentration of 50 µM, which completely blocks polar auxin transport in tobacco, greatly inhibited cell divisions and stimulated cell elongation, and caused a dramatic loss of file polarity [19]. In light-grown tomato seedlings, NPA inhibited root growth, but contrary to *Arabidopsis*, it stimulated hypocotyl elongation and epidermal cells of NPA-treated seedlings were more elongated and narrower than control seedlings [20]. These results suggested that in addition to cambial activity, cell division stimulation in cortex and pith tissues by NPA was induced in succulent plants by a local auxin accumulation caused by NPA application.

Relatively little information is available about the effect of NPA on cellular metabolism and endogenous auxins. As shown in Figure 3a and Figure S4a, NPA application to stems of *B. calycinum* and *B. daigremontianum* induced significantly increased levels of endogenous IAA at the site of its application, while this effect was not observed in the areas above and below the treatment. Ringing inflorescence stems with NPA of intact *Arabidopsis thaliana* inhibited stem radial development below the ringing site, as well as the development of fascicular xylem, interfascicular xylem and interfascicular extraxylary fibers; however, fascicular xylem production was enhanced in the area above the ringing [21]. Suer et al. [22] documented that NPA-induced auxin accumulation stimulated cambium activity in the stems of wild-type *Arabidopsis thaliana*, but not in WOX4 mutants, although basal cambial activity was not abolished. Ji et al. [23] and Hirakawa et al. [24] identified an important role of WOX4 transcription factor in promoting cambial activity. Thus, Suer et al. [22] have shown that *WOX4* is one of essential factors that make the cambium responsive to the long-distance regulation by auxin transported basipetally along the stem. Mattsson et al. [25] showed that plants (*Arabidopsis thaliana*, *Aurinia saxatile*, *Anthurium majus*, *Nicotiana tabacum*) grown on NPA-supplemented media formed inflorescence stems with increased width of fascicular xylem and interfascicular extraxylary fibers. These results suggest that local accumulation of IAA at the NPA application site in succulent plants stimulates conspicuous local stem swelling by influencing cambial activity and cell structures in vascular tissues. The mechanism of local accumulation of IAA at the site of NPA application has not been elucidated, but almost lack of inhibition of polar auxin transport, compared to TIBA and HFCA, or *de novo* synthesis of IAA in the treated area might be possibly involved.

While relatively little information is available about the effect of NPA on other plant hormones in relation to conspicuous local stem swelling, comprehensive analyses of plant hormones revealed that local accumulation of IAA and cytokinins was found in the NPA-treated area of *B. calycinum* and *B. daigremontianum* stems (Figure 3 and Figure S4, and Table 1 and Table S2). Cytokinins are known to be essential in promoting mitotic cell division and regulating cell cycle in the shoot, and they play a key role in organizing new shoot apical meristems [26,27]. Cytokinins stimulated stem diameter enlargement by promoting cell division in potato plants [28]. Shibaoka [29] documented that kinetin-induced stem thickening of light-grown epicotyl segments of Azuki bean was caused by changes in the orientation of cell wall microtubules from randomly oriented to parallel to the cell axis. Cytokinins have been demonstrated to induce a greater thickness of intact etiolated pea seedlings [30], and this effect was more pronounced the presence of auxin in stem segments of etiolated and light-grown pea seedlings, soybean seedlings, and others [31,32,33,34]. After removing all leaves and flower buds in tulip plants, the application of IAA at a concentration of 0.1 and 2.0% (*w/w* in lanolin) to the decapitated site greatly restored stem growth, while the additional application of benzyladenine (BA) to the tulip stem by soaking a cotton wick wrapped around all internodes, significantly stimulated thickening of all internodes [35]. NPA application mimicked cytokinin induction of the off-the-medium growth of *Arabidopsis* root tip [36], as well as the effect of exogenous cytokinin in inducing root-like organogenesis in excised *Arabidopsis* hypocotyls [37]. Hu et al. [38] indicated that NPA also enhanced shoot organogenesis in citrus epicotyl explants, independent of its involvement in auxin transport, and demonstrated that the promotional effect of NPA on shoot organogenesis was cytokinin-dependent. Interestingly, BA at a concentration of 0.2% also induced local stem swelling in intact *B. calycinum* to a degree similar to NPA treatment (Figure S5); however, BA did not induce swelling in decapitated internode of *B. calycinum*. In stem mustard (*Brassica juncea* var. tsatsai cultivar), the contents of zeatins, including zeatin ribosides and IAA have been demonstrated to correlate with stem swelling induced by ambient (or relatively low) temperature [13]. These facts suggest that cytokinin effect on conspicuous local stem swelling is closely related to local auxin accumulation in *B. calycinum*. Local NPA action mechanism in the stem might be based on a response involving crosstalk between the auxin and cytokinin regulatory pathways.

The application of NPA also induced changes in endogenous levels of JA and its precursor, 12-oxo-PDA, in the swollen part of the stem induced by NPA (Figure 3 and Figure S4, Table 1 and Table S2). Jasmonates (JAs), referred to as jasmonic acid and its related compounds, have been demonstrated to exhibit tuber-inducing activity in potato stolons [39]. In isolated tomato roots cultured in vitro, JA induced swollen root apices and increased cellular vacuolation, and JA did not act directly through ethylene [40]. A close functional relationship between the JA signaling pathway and auxin homeostasis has been also documented [41,42,43,44]. These results suggest that the NPA-induced increase in endogenous levels of JAs and its interaction with IAA contribute to the regulation of conspicuous local stem swellings in *Bryophyllum* plants.

Further research explaining the mechanism of NPA-induced local accumulation of IAA and cytokinins as well as JAs will be required in the near future.

## 4. Materials and Methods

### 4.1. Plant Materials and the Application of NPA and TIBA

*Bryophyllum calycinum* plants (4–6-month-old) propagated from the buds formed in the marginal notches of excised leaves and grown under natural light conditions and temperature in a glasshouse were used for the experiments.

The plants were treated with *1*-naphthylphthalamic acid [(NPA, 0.4%, *w/w*)] (Sigma-Aldrich Ltd., St. Louis, MO, USA) or 2,3,5-triiodobenzoic acid [(TIBA, 0.4%, *w/w*)] (Sigma-Aldrich Ltd., St. Louis, MO, USA) in a lanolin paste as a 2-mm-wide ring around the stem in the internode with intensive growth. Plants treated with lanolin only were used as control. For histological analyses, the experiments were repeated at least four times with 10 to 15 plants, and the experiments involving comprehensive analyses of endogenous plants hormones, were carried out using 25 plants for NPA treatment and lanolin treatment only (control), respectively.

In some experiments, 4- to 6-month-old *B. daigremontianum* and *Kalanchoe tubiflora* plants propagated from naturally formed plantlets on leaves and grown under natural light conditions and temperature in a glasshouse were used. *Sedum spectabile*, an ornamental herbaceous perennial plant of the family Crassulaceae, growing outside under natural conditions in a garden or in a glasshouse at The National Institute of Horticultural Research, Skierniewice, Poland in the period from early July to September was also used.

### 4.2. Histological Analyses of Conspicuous Local Stem Swelling in Succulent Plants

Histological analyses of conspicuous local stem swelling induced by 0.4% NPA in *B. calycinum* at the site of NPA application and the areas above and below were made 8 days after treatment. For histological observation, 0.7-cm stem pieces were excised from the swollen areas induced by NPA, and regions above and below the NPA treatment in the same plants; corresponding fragments from untreated plants were used as controls. Five samples of internode segments were collected from each shoot.

The materials were fixed in a solution of chromic acid, acetic acid, and formalin (CrAF) for 48 h at room temperature, dehydrated through an increasing alcohol series (70%, 80%, 90% and 100%), and embedded in paraffin according to the method reported previously [45]. Transverse and longitudinal sections (12-μm-thick) were cut with a rotary microtome (Leica, Wetzelar, Germany) and stained with safranin (1% prepared in ultrapure water) and fast green (1% prepared in 95% ethanol). The sections were mounted in Canada balsam and analyzed using a light microscope (Eclipse 80i, Nikon, Tokyo, Japan) with NIS-Elements BR ver. 4.00 imaging software (Nikon Instruments Inc., Tokyo, Japan) for photo documentation.

Similar histological analyses of conspicuous local stem swelling induced by 0.4% NPA were also carried out in the stems of *K. tubiflora* and *S. spectabile.*

### 4.3. Comprehensive Analyses of Endogenous Plant Hormones in Succulent Plants

Eight days after treatment with NPA and lanolin only of *B. calycinum* plants, 4- to 5-mm internode fragments were excised in the swollen area and the areas above and below it with or without NPA treatment. The samples were frozen, lyophilized, and powdered.

Analyses of plant hormones were performed according to the methods reported previously [46,47,48,49]. The samples were spiked with a stable isotope-labeled internal standard mixture and extracted in methanol/water/formic acid solution (15/4/1, *v/v/v*). The obtained extract was evaporated and re-suspended 1 M formic acid with 3% of methanol. Samples underwent SPE (BondElut Plexa PCX, Agilent, Santa Clara, CA, USA) clean up as described previously [45,46]. Plant hormones analyses were done in triplicate on a UHPLC-MS/MS system (Agilent Infinity 1260, coupled to a 6410 Triple Quad LC/MS, Agilent, Santa Clara, CA, USA). Analyses were done in positive electrospray ionization (ESI) in multiple reaction monitoring (MRM) mode. As internal standards, [^15^N_4_]dihydrozeatin, [^2^H_5_]*trans*-zeatin riboside, [^2^H_5_]indole-3-acetic acid, [^2^H_4_]salicylic acid, [^2^H_2_]gibberellin A_1_, [^2^H_2_]gibberellin A_4_, [^2^H_2_]gibberellin A_5_, [^2^H_2_]gibberellin A_6_, [^2^H_6_]*cis, trans*-abscisic acid, [^2^H_5_]benzoic acid (OlChemim, Olomunc, Czech Republic), [^2^H_5_]jasmonic acid (CND Isotopes, QC, Canada), and [^2^H5]*dinor*-12-oxo-phytodienoic acid (Cayman Chem. Comp., Ann Arbor, MI, USA) were used. The technical details are given in the cited references.

Similar analyses of plant hormones in relation to conspicuous local stem swelling induced by 0.4% NPA were carried out in *B. daigremontianum* stems.

### 4.4. Determination of Polar Auxin Transport in Bryophyllum Calycinum Stem Segments

Following the method reported previously [9,10], with minor modifications, a system for the detection of bioassay-derived polar auxin transport was introduced consisting of 50 μL and 150 μL of 100-fold diluted [^14^C]-IAA with a specific activity of 55 mCi/mmol, 0.1 mCi/mL, (American Radiolabeled Chemicals Inc., St. Louis, MO, USA) at the bottom of 2-mL Eppendorf tubes (for 25 mm stem segments) and 6-mL plastic vials (for 40-mm stem segments), respectively.

Internode segments (25 mm in length) excised from growing internodes were used, mainly from the second or third internode from the top of growing plants with active elongation. NPA and TIBA (0.4% *w/w*) were applied in lanolin as a 2-mm strip to the middle part around the internode segments. Internode segments treated only with lanolin served as the control segments. In another experiment, internode segments (40 mm in length) prepared from the growing internode of intact plants pretreated with NPA or TIBA for 24 h were used to determine polar auxin transport.

The 20- and 40-mm-long segments were placed into plastic Eppendorf tubes and vials, respectively, facing down the apical side. There was almost no auxin transport observed when IAA was applied at the basal side of the segments in the same manner (data not shown). Therefore, ^14^C-labelled IAA applied to the apical side was transported to the physiological apical–basal direction in this assay system.

After incubation at 23.5 °C for 18 h, a 3-mm piece was cut on the opposite side of the segment. The piece was directly put into a vial containing liquid scintillation cocktails (Universol^TM^-ES, MP-Biomedicals, CA, USA), and then, its radioactivity was determined using a liquid scintillation counter (Tri-Carb2200CA, Packard Instrument Co., Ltd., Meriden, CT, USA). The experiments were performed in triplicate with 6 segments. The results are expressed as percentages of the mean control values with standard errors (*n* = 3).

### 4.5. Statistical Analysis

The analysis of variance (ANOVA) was conducted using STATISTICA software (StatSoft, Kraków, Poland). When ANOVA indicated significant effects, the means were separated using Duncan’s multiple range test or Student’s *t*-test at *p* < 0.05 considered to be statistically significant.

## 5. Conclusions

Unlike TIBA and HFCA, NPA shows unusual physiological effects that differed from other polar auxin transport inhibitors in succulent plants; NPA induced conspicuous local stem swelling in intact plants of *B. calycinum* and some other succulent plants as well as rooting of shoot cuttings. NPA showed almost no inhibition of polar auxin transport in *B. calycinum* plants. NPA also induced a significant increase in the levels of IAA, cytokinins, and JA and its precursor, 12-oxo-PDA, at its application site in succulent plants. The crosstalk of these plant hormones might regulate cell division and enlargement, resulting in conspicuous local stem swelling. Further studies on the mechanism of NPA induced local accumulation of these plant hormones will be required in the near future.

## Figures and Tables

**Table 1:** Effect of NPA (0.4%, *w/w*) on endogenous levels of abscisic acid, salicylic acid, *cis*-zeatin, *cis*-zeatin riboside, and gibberellins determined in 4–5-mm stem pieces treated with or without NPA in *B. calycinum.* Above and below in the table indicate plant hormone levels in the above and the below areas of the treatment, respectively. Values are the means with standard errors (*n* = 3). Different letters indicate a statistical difference in Duncan’s multiple range test at *p* < 0.05 after ANOVA.

Plant Hormone, ng/gDW	Control (Lanolin)			NPA Treatment		
	Above	Treated Area	Below	Above	Treated Area	Below
Abscisic acid	163.3 ± 2.9 b	204.9 ± 6.2 b	211.0 ± 4.8 b,c	209.6 ± 3.7 a	260.1 ± 17.0 a	239.7 ± 5.7 c
Salicylic acid	1509 ± 157 b,c	1520 ± 134 a,b,c	2254 ± 288 a	2332 ± 70 b,c	4631 ± 787 c	1667 ± 106 a,b
*cis*-Zeatin	5.5 ± 0.17 a	7.1 ± 0.16 c	6.2 ± 0.07 b	6.8 ± 0.22 b,c	8.7 ± 0.27 d	7.1 ± 0.07 c
*cis*-Zeatin riboside	0.1 ± 0.06 a	0.2 ± 0.16 a	0.0 ± 0.01 a	0.5 ± 0.16 a	1.2 ± 0.0.37 b	0.5 ± 0.12 a
Gibberellin A_1_	13.8 ± 0.8 c	18.1 ± 1.4 b	23.5 ± 1.1 c	14.6 ± 1.2 a	20.9 ± 0.9 a	21.7 ± 1.3 c
Gibberellin A_4_	5.0 ± 1.0 a	6.7 ± 0.9 a	6.3 ± 1.8 a	9.4 ±1.8 a	9.7 ± 2.7 b	8.4 ± 2.7 a
Gibberellin A_19_	35.5 ± 1.9 b	44.7 ± 0.7 a	48.9 ± 0.8 a	53.5 ± 0.3 b	47.4 ± 2.9 b	52.5 ± 2.7 b
Gibberellin A_44_	3.0 ± 0.1 b	4.1 ± 0.1 b	5.1 ± 0.2 a	2.5 ± 0.2 b	4.8 ± 0.5 b	3.8 ± 0.4 b
Gibberellin A_15_	1.4 ± 0.1 b	9.2 ± 0.2 a	1.1 ± 0.1 b	1.6 ± 0.1 b	1.2 ± 0.5 a	2.5 ± 0.2 a
Gibberellin A_53_	21.5 ± 2.1 b	54.2 ± 4.7 c	106.7 ± 2.6 c	55.6 ± 2.6 a	63.3 ± 14.3 d	129.8 ± 9.7 b,c
Gibberellin A_9_	35.3 ± 2.7 a,b	43.7 ± 0.9 b,c	41.0 ± 5.7 a	39.4 ± 1.6 d	40.3 ± 3.2 c	49.2 ± 1.6 b,c
Gibberellin A_8_	50.7 ± 11.7 a,b	64.9 ± 29.6 b,c	72.9 ± 8.2 a	72.1 ± 9.9 c	79.2 ± 16.8 c	76.0 ± 18.8 a

## Data Availability

Not applicable.

## Supplementary Materials

Supplementary Materials can be found at https://www.mdpi.com/1422-0067/22/6/3118/s1.
