# Supplementary material for: Mode of Action of 1-Naphthylphthalamic Acid in Conspicuous Local Stem Swelling of Succulent Plant, Bryophyllum calycinum: Relevance to the Aspects of Its Histological Observation and Comprehensive Analyses of Plant Hormones
Source: Int J Mol Sci. 2021 Mar 18;22(6):3118. doi: 10.3390/ijms22063118 (PMC8003132; doi:10.3390/ijms22063118)
Supplement: Supplementary file 1 [file ijms-22-03118-s001.pdf]

Supplementary material for:

Mode of Action of *1*-Naphthylphthalamic Acid in Conspicuous Local Stem Swelling of Succulent Plant, *Bryophyllum calycinum*: Relevance to the Aspects of Its Histological Observation and Comprehensive Analyses of Plant Hormones

Agnieszka Marasek-Ciolakowska [1,*,†], Michał Dziurka [2,†], Urszula Kowalska [1], Justyna Góraj-Koniarska [1], Marian Saniewski [1], Junichi Ueda [3] and Kensuke Miyamoto [4,*]

[1] The National Institute of Horticultural Research, Konstytucji 3 Maja 1/3, 96-100 Skierniewice, Poland; urszula.kowalska@inhort.pl (U.K.); justyna.goraj@inhort.pl (J.G.-K.); marian.saniewski@inhort.pl (M.S.)

[2] The Franciszek Górski Institute of Plant Physiology, Polish Academy of Sciences, Niezapominajek 21, 30-239 Kraków, Poland; m.dziurka@ifr-pan.krakow.pl

[3] Department of Biological Science, Graduate School of Science, Osaka Prefecture University, 1-1 Gakuen-cho, Naka-ku, Sakai, Osaka 599-8531, Japan; ueda@b.s.osakafu-u.ac.jp

[4] Faculty of Liberal Arts and Sciences, Osaka Prefecture University, 1-1 Gakuen-cho, Naka-ku, Sakai, Osaka 599-8531, Japan

* Correspondence: agnieszkamarasek@wp.pl; Tel.: +48-46-8346783 (A.M.-C.); miyamoto@las.osakafu-u.ac.jp; Tel. +81-72-254-9741 (K.M.)

† These authors contributed equally to this work.

Table S1. Effects of NPA, TIBA and HFCA on rooting of *Sedum spectabile* shoot cuttings.

The effect was shown as fresh weight (mg) of roots of rooting shoot cuttings. Young shoot cuttings with length of 15 cm were used. Each polar auxin transport inhibitor was applied as a ring (2 mm width) around the lower side of the stem under all leaves of the cuttings, and then the treated cuttings were subjected to the rooting system; keeping for 30 days in water in 100 ml Erlenmeyer flasks with change of water every three days under room conditions and for 43 days in soil medium in greenhouse under natural conditions. At the end of experiment weight of roots was measured. Five cuttings were used for each treatment and the experiments were repeated twice. The results were statistically analyzed by one-way analysis of variance (ANOVA) using the STATISTICA program (13.1 PL 2012, StatSoft, Poland). Different letters indicate statistically significant at the $P<0.05$ levels according to the Duncan's multiple range test.

Treatment	Rooting in soil medium, mg /shoot cutting	Rooting in water, mg/shoot cutting
Control (lanolin paste)	883 c	369 b
NPA (0.4%, w/w in lanolin)	851 c	406 b
TIBA (0.2%, w/w in lanolin)	74 b	0 a
HFCA (0.2%, w/w in lanolin)	0 a	0 a

Table 2. Effect of NPA (0.4%, w/w) on endogenous levels of abscisic acid, salicylic acid *cis*-zeatin, *cis*-zeatin riboside and gibberellins identified in 4-5 mm stem pieces treated with or without NPA in *Bryophyllum daigremontianum.* Values are the mean with standard errors (n=3). Different letters indicate statistic difference by Duncan's multiple range test with $P<0.05$ after ANOVA. Others including growth experiment were the same as *B. calycinum* as shown in Table 1.

**Plant hormone, ng/gDW**	**Control (lanolin)**			**NPA Treatment**		
	**above**	**treated area**	**below**	**above**	**treated area**	**below**
Abscisic acid	501.8 ± 5.5 b	496.2 ± 23.6 b	557.9 ± 44.6 bc	359.1 ± 26.4 a	416.7 ± 7.0 a	621.6 ± 16.1 c
Salicylic acid	16325 ± 3328 bc	10506 ± 1182 abc	6251 ± 521 a	16284 ± 2048 bc	17561 ± 4954 c	8078 ± 818 ab
cis-Zeatin	6.7 ± 0.10 c	5.8 ± 0.07 ab	6.5 ± 0.12 c	5.4 ± 0.17 a	5.7 ± 0.14 ab	6.0 ± 0.18 b
cis-Zeatin riboside	0.5 ± 0.06 a	0.7 ± 0.05 a	0.7 ± 0.07 a	0.9 ± 0.09 ab	2.2 ± 0.17 c	1.1 ± 0.16 b
Gibberellin A_1	23.3 ± 0.7 c	17.8 ± 0.5 b	26.2 ± 3.3 c	12.9 ± 0.4 a	11.9 ± 0.3 a	24.5 ± 0.6 c
Gibberellin A_4	5.2 ± 0.3 a	6.2 ± 0.2 a	4.5 ± 0.4 a	5.2 ± 0.6 a	9.7 ± 1.9 b	4.5 ± 0.4 a
Gibberellin A_{19}	49.7 ± 2.8 b	39.7 ± 2.7 a	59.5 ± 2.9 c	40.6 ± 1.4 a	44.7 ± 0.9 ab	61.3 ± 2.7 c
Gibberellin A_{44}	12.6 ± 0.2 b	14.6 ± 1.8 b	9.0 ± 0.6 a	13.4 ± 0.8 b	13.5 ± 0.3 b	13.6 ± 0.6 b
Gibberellin A_{15}	1.6 ± 0.1 b	0.5 ± 0.2 a	1.4 ± 0.1 b	1.2 ± 0.1 b	0.4 ± 0.2 a	0.5 ± 0.2 a
Gibberellin A_{53}	33.6 ± 3.7 b	46.3 ± 3.6 c	51.1 ± 3.9 c	13.7 ± 2.8 a	69.3 ± 3.9 d	41.4 ± 2.0 bc
Gibberellin A_9	54.4 ± 6.1 ab	66.2 ± 5.8 bc	45.6 ± 4.7 a	102.2 ± 5.8 d	76.1 ± 5.2 c	69.6 ± 3.5 bc
Gibberellin A_8	271.3 ± 26.1 ab	559.0 ± 98.9 bc	118.9 ± 59.0 a	878.2 ± 271.0 c	679.5 ± 56.7 c	82.0 ± 29.9 a

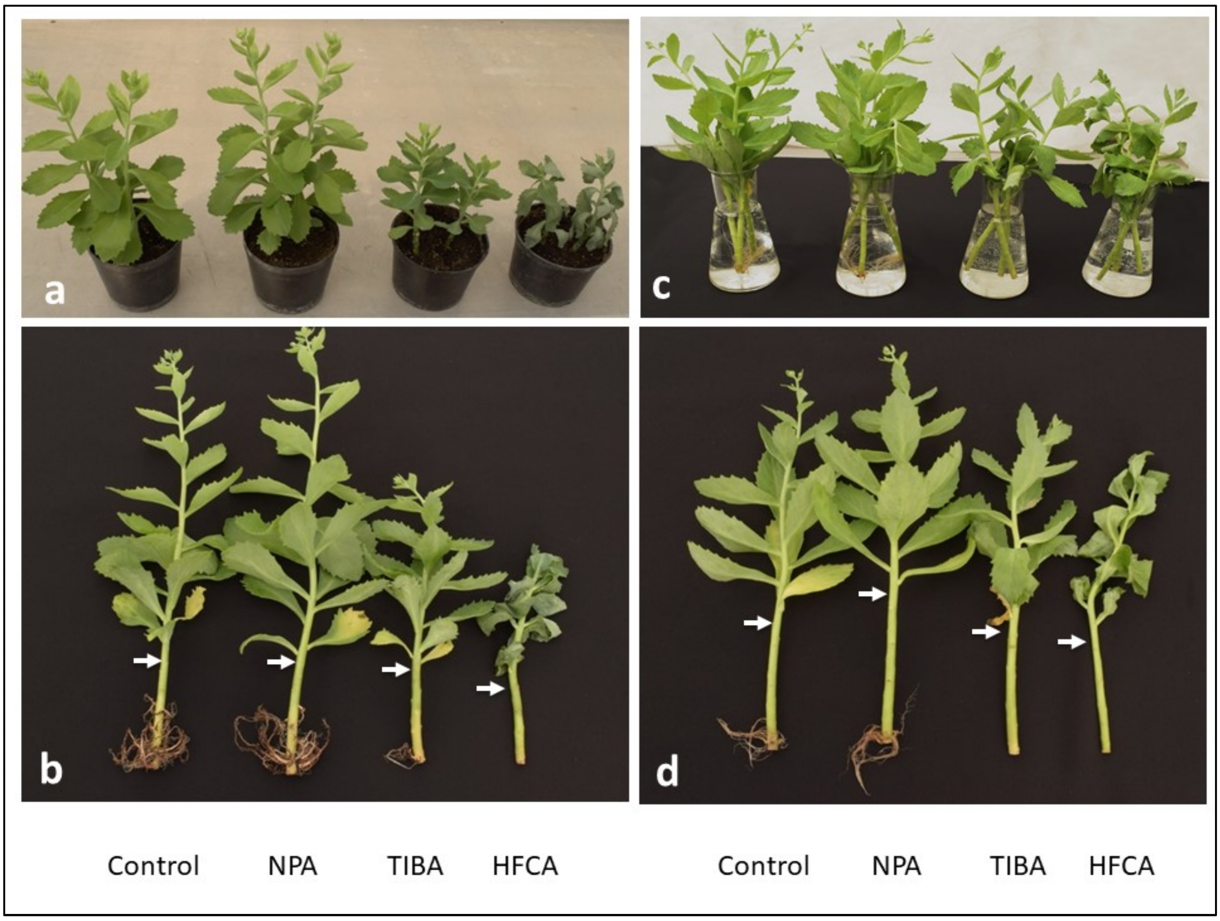

Figure S1. Effects of NPA, TIBA and HFCA on rooting of *Sedum spectabile* shoot cuttings. Young shoot cuttings with length of 15 cm were used. Each polar auxin transport inhibitor (0.2%, w/w in lanolin) was applied as a ring (2 mm width) around the lower side of the stem under all leaves of the cuttings, and then the treated cuttings were subjected to the rooting system; keeping for 30 days in water in 100 ml Erlenmeyer flasks with change of water every three days under room conditions and for 43 days in soil medium in a greenhouse under natural conditions. **a** and **b**: rooting of shoot cuttings in soil after 43 days; **c** and **d**: rooting of shoot cuttings in water after 30 days. Arrows indicate the treated area.

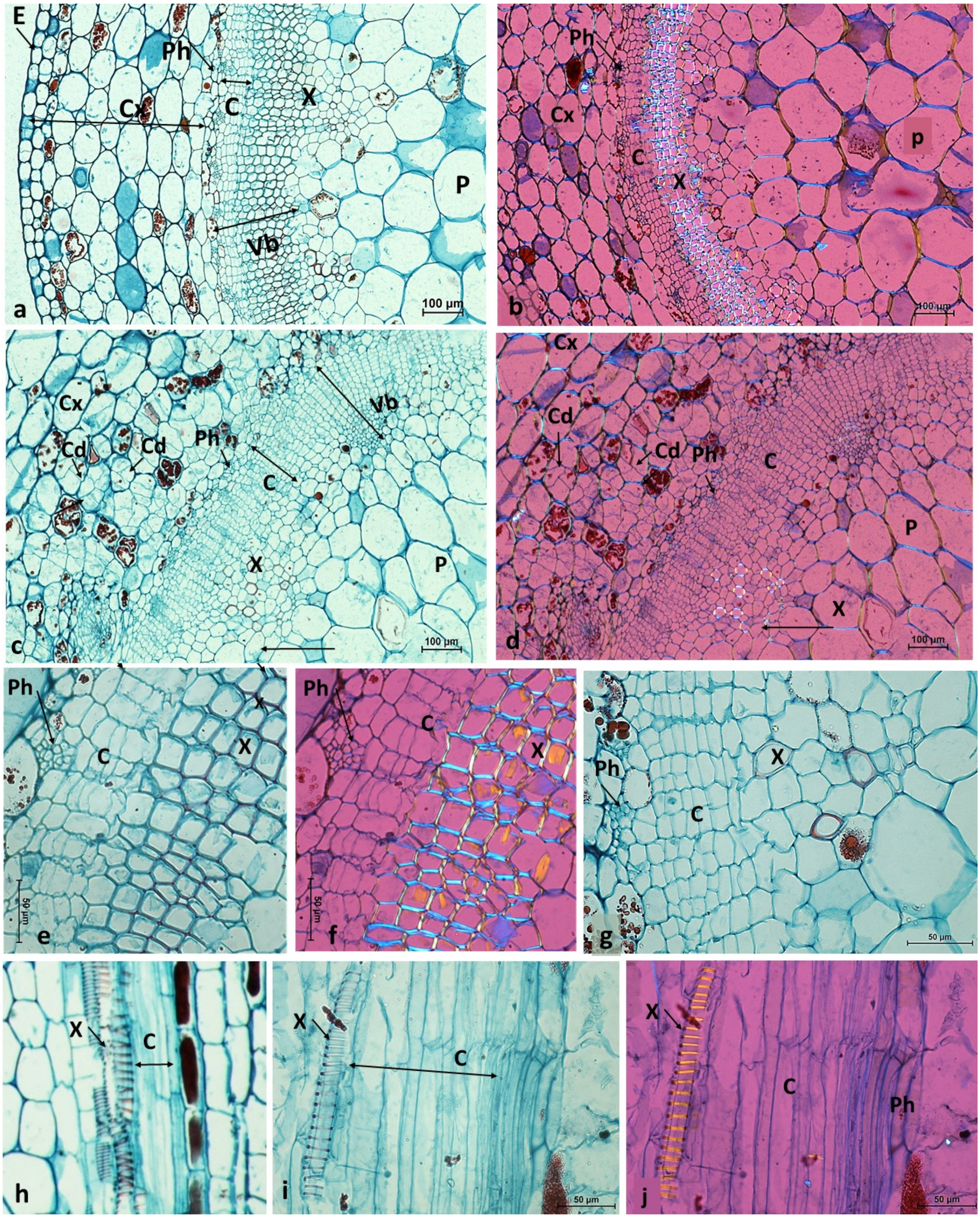

Figure S2. Anatomical details in the middle of internode of *Kalanchoe tubiflora.* **a** and **b**: Cross-section of the stem treated with lanolin only in light microscope (LM) (**a**), and light microscope with polarization light (LMP) (**b**). **c and d:** Cross-section of the stem at the place of swelling induced by NPA at 0.4% (w/w in lanolin) in light microscope (LM) (**c**), and light microscope with polarization light (LMP) (**d**). Note numerous cell divisions (Cd) in cortex were observed. **e and g**: Details of vascular tissue on cross section of control stem in LM (**e**) and LMP (**f**), and of swelling stem induced by the application of NPA in LM (**g**). **h-j**: Details of vascular tissue on longitudinal section in untreated stem (control) in LM (**h**), and swelling stem induced by NPA at 0.4% in LM (**i**) and in LMP (**j**). C: cambium; Cd: cell division; Cx: cortex; E: epidermis; P: pith; Ph: phloem; VB: vascular tissues; X: xylem.

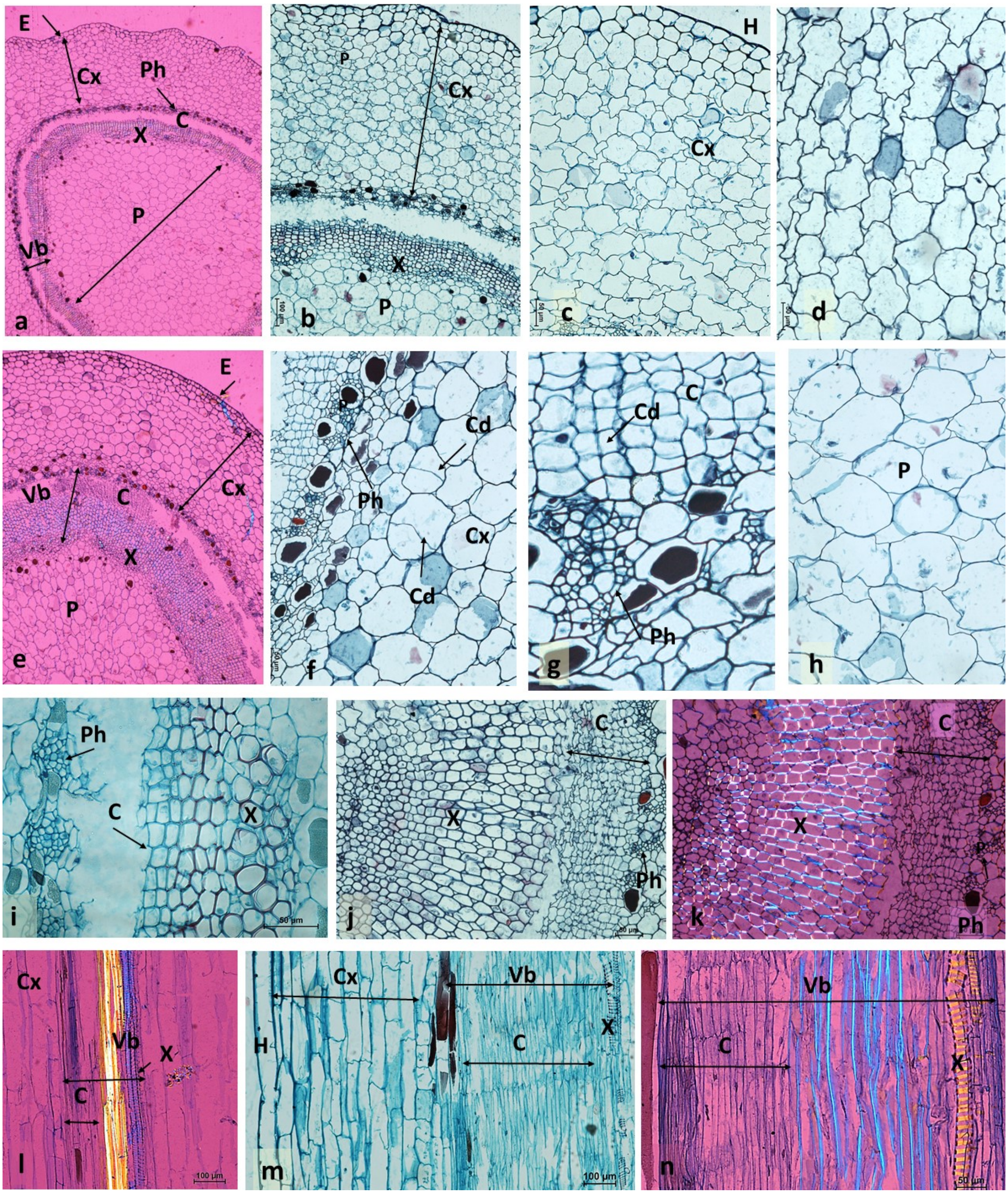

Figure S3. Anatomical details of the middle of internode of *Sedum spectabile*. **a-d**: Cross-section of the stem treated with lanolin only in light microscope with polarization light (LMP) (**a**), and light microscope (LM) (**b-d**). **c** and **d**: Details of cortex (**c**) and pith in LM (**d**). **e-h**: Cross-section of stem swelling induced by NPA at 0.4% (w/w in lanolin) in LMP (**e**) and in LM (**f-h**). **f** and **h**: Details of cortex (**f**), vascular tissues (**g**), and pith in LM (**h**). **i**: Details of vascular tissue on cross-section in LM in control plant. **J** and **k**: Details of the vascular tissues on cross-section in stem swelling induced by NPA in LM (**j**) and in LMP (**k**). **l-n:** Longitudinal section of the vascular tissue and cortex in control stem in LMP (**l**), and in stem swelling induced by NPA at 0.4% in LM and LMP. C: cambium; Cd: cell division; Cx: cortex; E: epidermis; P: pith; Ph: phloem; VB: vascular tissues; X: xylem.

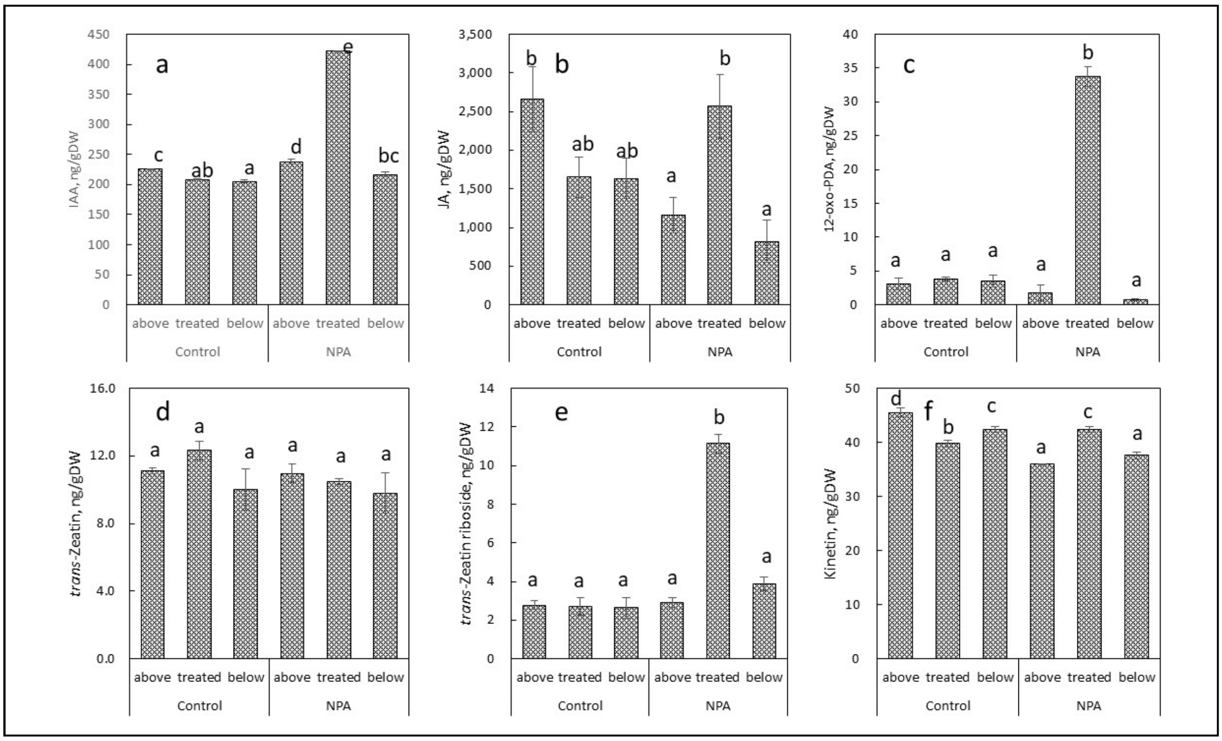

Figure S4. Effect of NPA (0.4%, w/w in lanolin) on endogenous levels of IAA (a), jasmonic acid (JA) (b), 12-oxo-phytodienoic acid (12-oxo-PDA) (c), *trans*-zeatin (d), *trans*-zeatin riboside (e) and kinetin (f) in 4-5 mm pieces of the stem treated with or without NPA in *Bryophyllum daigremontianum*. Treated, above and below in the figure indicate plant hormone levels at, above and below areas of the treatment respectively. Values are the mean with standard errors (n=3). Different letters indicate statistic difference by Duncan's multiple range test with $P<0.05$ after ANOVA. Others including growth experiment were the same as *B. calycinum* as shown in Fig. 3.

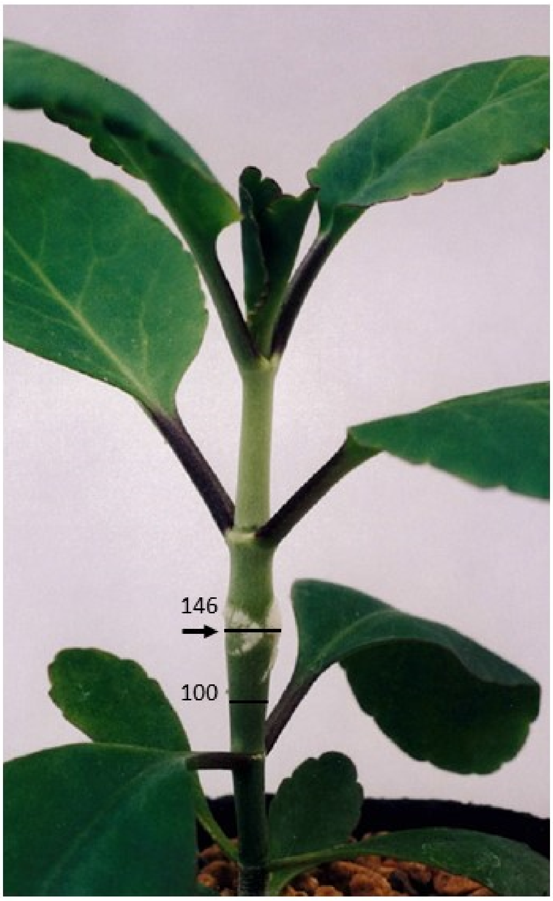

Figure S5. Effect of benzyladenine (BA, 0.2% w/w in lanolin) on stem swelling of *B. calycinum* after 7 days of the treatment. Arrow indicates the place of BA application as a 2-mm-wide ring of lanolin paste around the stem in the intensively growing internode. Value with lines in the photograph indicates relative diameter (%) of the non-treated area (100%).
